# Skill Level in Tennis Serve Return Is Related to Adaptability in Visual Search Behavior

**DOI:** 10.3389/fpsyg.2021.689378

**Published:** 2021-09-20

**Authors:** Jernej Rosker, Ziva Majcen Rosker

**Affiliations:** ^1^Faculty of Health Sciences, University of Primorska, Koper, Slovenia; ^2^Faculty of Sport, University of Ljubljana, Ljubljana, Slovenia

**Keywords:** expertise, racquet sports, interception tasks, focal vision, visual fixations

## Abstract

Analyzing visual search strategies in tennis is primarily focused on studying relationships between visual behavior and tennis performance. However, diverse movement characteristics among different servers suggest the importance of adjusting the visual search strategies of an individual while playing against different opponents. The aim of this study was to analyze whether visual search strategies can be attributed to the individual server and the returning player during the tennis serve return or return performance. Seventeen tennis players were enrolled in this study (five international players and 12 national players) producing a sample of 1,020 returns measured with mobile eye trackers. The random forest machine learning model was used to analyze the ability to classify the returning player [area under the curve (AUC): 0.953], individual server (AUC: 0.686), and return performance category (AUC: 0.667) based on the location and duration of the focal vision fixation. In international tennis players, the higher predictability of the server was observed as compared with national level players (AUC: 0.901 and 0.834, respectively). More experienced tennis players presented with a higher ability to adjust their visual search strategies to different servers. International players also demonstrated anticipatory visual behavior during the tossing hand movement and superior information pickup during the final phases of the stroke of a server.

## Introduction

Efficient visual search strategies during interceptive precision tasks in highly dynamic sports have been associated with superior sports performance. Among others, they enable more accurate anticipation of forthcoming events, fast and accurate decision-making, and online movement adaptation (Triolet et al., 2013; Woolley et al., 2015; Connor and Knierim, 2017). However, studies suggest high interindividual and intraindividual variability in visual search patterns making it difficult to fully understand their function and adaptability (Dicks et al., 2017).

Several factors have been proposed to contribute to the characteristics of visual search patterns and their variability, such as temporal and spatial demands of the task, amount of information available during the task performed, and knowledge about visual properties and regularities of the environment (Paeye and Madelain, 2014; Dicks et al., 2017). In interceptive tasks, such as returning tennis serve, saving a penalty kick in soccer, or making a save in field hockey, intercepting a ball is performed under temporal and spatial constraints and requires movement preparation, execution, and adaptation in a time window that can exceed the action of an opponent and the travel time of a ball (Jackson and Mogan, 2007; Müller and Abernethy, 2012; Morris-Binelli et al., 2021). Three main phases during interception tasks have been proposed to contribute significantly to interception performance (Müller and Abernethy, 2012; Mecheri et al., 2019). In the first preflight phase, the movement characteristics of an opponent are perceived, allowing the observer to initiate his movement toward the interception point. In the second phase, early ball flight characteristics are perceived and used to guide the interception movement, and in the third phase, late ball flight information is used to fine-tune the interception movement. Therefore, the role of specific visual information varies between different phases of interception tasks and contributes specifically to movement preparation and execution (Müller and Abernethy, 2012).

In addition, the movements of opponents are characterized by inherent movement variability (Latash, 2010), which poses a challenge for observers to extract relevant visual information. Opponents produce different visual cues by applying phase-specific synergies of limb and body movements (Maselli et al., 2017; Shafizadeh et al., 2019). These synergies change depending on the phase of the movement of an opponent. Such noisiness in stimulus presentation inherently leads to higher variability in the visual search strategies employed by the observer (Paeye and Madelain, 2014). For example, in the tennis serve return, the highest variability in the upper limb movements of a server was associated with the preparation phase of the serve and the ball contact phase. Between these two phases, the vertical ball toss was identified as one of the most critical phases related to anticipation of serve type and movement initiation (Jackson and Mogan, 2007; Mecheri et al., 2019). The movements of the arm and racquet during the backswing and upswing are thought to contribute to the anticipation of ball flight direction and guide the initial movements of the returning player (Jackson and Mogan, 2007; Button et al., 2011; Navia et al., 2017).

A considerable body of literature has demonstrated differences in visual behavior between more and less experienced athletes (Jackson and Mogan, 2007; Land, 2009; Button et al., 2011; Lebeau et al., 2016; Murray and Hunfalvay, 2017). More experienced athletes are able to use fewer saccades and longer fixations than less-skilled counterparts, as well as earlier fixations relative to the interception movement termination phase. In addition, their visual search strategies rely more on the top-down control of attention (Aglioti et al., 2008). This allows higher-skilled athletes to use more consistent visual search strategies while observing noisy opponent preflight actions compared with less skilled observers (Jones and Miles, 1978; Aglioti et al., 2008; Müller and Abernethy, 2012).

An additional factor affecting the characteristics of eye movements and consequently their variability is the type of research protocol used. Video-based observational studies are commonly used (Murray and Hunfalvay, 2017), which can be augmented with video occlusion techniques (Farrow et al., 2005; Mecheri et al., 2011; Giblin et al., 2017). However, these studies have been criticized as attending to visual cues and anticipating ball flight direction from a video recording are thought to differ in underlying neural processes as compared with *in vivo* measures of visual attention (Dicks et al., 2010). More specifically, the ventral visual information processing pathway is involved in video-based experiments, and the dorsal visual processing pathway is involved in real game situations (Müller and Abernethy, 2012). This has important implications for conducting ecologically valid studies. In other words, the dorsal pathway is responsible for rapid visual perception and its connection to movement, whereas the ventral pathway involves cognitive processes for recognizing objects of interest (Vaziri-Pashkam and Xu, 2017). Moreover, video-based studies usually present visual stimuli, which are deprived of at least some types of auditory stimuli. As reported in the literature, the presence of multisensory stimuli may reduce the variability of visual search strategies (Murray et al., 2019). In addition, the motor experience of an observer in performing the observed movement patterns contributes significantly to visual behaviors, such as a lower number of rapid saccadic eye movements and longer fixation times (Aglioti et al., 2008). Moreover, advanced anticipation in expert players has been shown to correlate with increased cortical activity, especially in areas within the medial and lateral frontal cortex which are critical for observing and interpreting actions performed by opponents (Wright et al., 2010). Overall, these factors may contribute to the noisiness of visual search patterns when searching for relevant visual information.

Although differences between athletes of different skill levels and successful and less successful task performance have been well-documented, no systematic attempts have been made to analyze other possible sources of variability in a visual search pattern, such as observing different opponents. This is particularly relevant given that in sports, opponents change regularly and present different subject-specific constraints. For example, kinematics related to ball flight direction have been shown to be specific to individual throwers, with high intra-thrower variability (Maselli et al., 2017). This in itself presents a particular challenge for the observer to extract opponent-specific relevant visual information.

The noisiness of visual search strategies poses a specific methodological challenge to study the different aspects of visual attention. To better understand the characteristics of visual perception in the context of sports performance, some authors (Dicks et al., 2017) have proposed the use of non-linear analysis methods. Such computational approaches allow the identification of other factors that contribute to sports performance, such as the adaptability of visual behavior to different opponents.

Therefore, the aim of this study was to apply a robust data mining method to investigate whether visual search strategies during the tennis serve return are related to the individual opponent (individual server), observer (returning player), and tennis serve return performance. We hypothesized that visual fixation duration and its position on the server during different phases of tennis serve return would be related to the individual returning player but to a lesser extent to the specific server and serve-return performance, regardless of the quality level of a player which was the primary goal of this study. The secondary goal was to investigate whether new characteristics in the aforementioned visual attention behavior could be identified that may contribute to a better understanding of return performance and direct future research.

## Materials and Methods

### Participants

Seventeen male tennis players who were enrolled in this study were divided into two groups. Twelve tennis players competing at a national level (mean age 20.0 ± 1.5 years, height 1.80 ± 0.06 m, weight 77.1 ± 6.7 kg, training experience 13 ± 0.8 years, all subjects were right-handed, placed in the top 20 players at the national junior rankings) were included in the first group. Four professional players competing at Davis Cup and one former professional international tennis player (mean age 30.5 ± 2.8 years, height 1.80 ± 0.08 m, weight 78.2 ± 6.3 kg, training experience 19 ± 4.7 years, one subject was left-handed) were included in the second group. Two players in the second group were ranked between the top 350 players and two players between the top 900 players in the ATP singles ranking. One player was retired, ranked between the top 300 players in the ATP singles ranking 1 year before the experiment but was still highly active as a tennis player. All players were contacted directly and invited to participate in this study. Subjects had to be enrolled in training that consisted of more than five training sessions per week, were free of musculoskeletal injuries 6 months before the experiment, and had a normal or corrected-to-normal vision. All participants were required to read and sign an informed consent form. This study was approved by the National Committee for Medical Ethics (No. 0120-47/2020/6) and was conducted according to the Declaration of Helsinki.

### Design and Procedure

**Figure 1:**
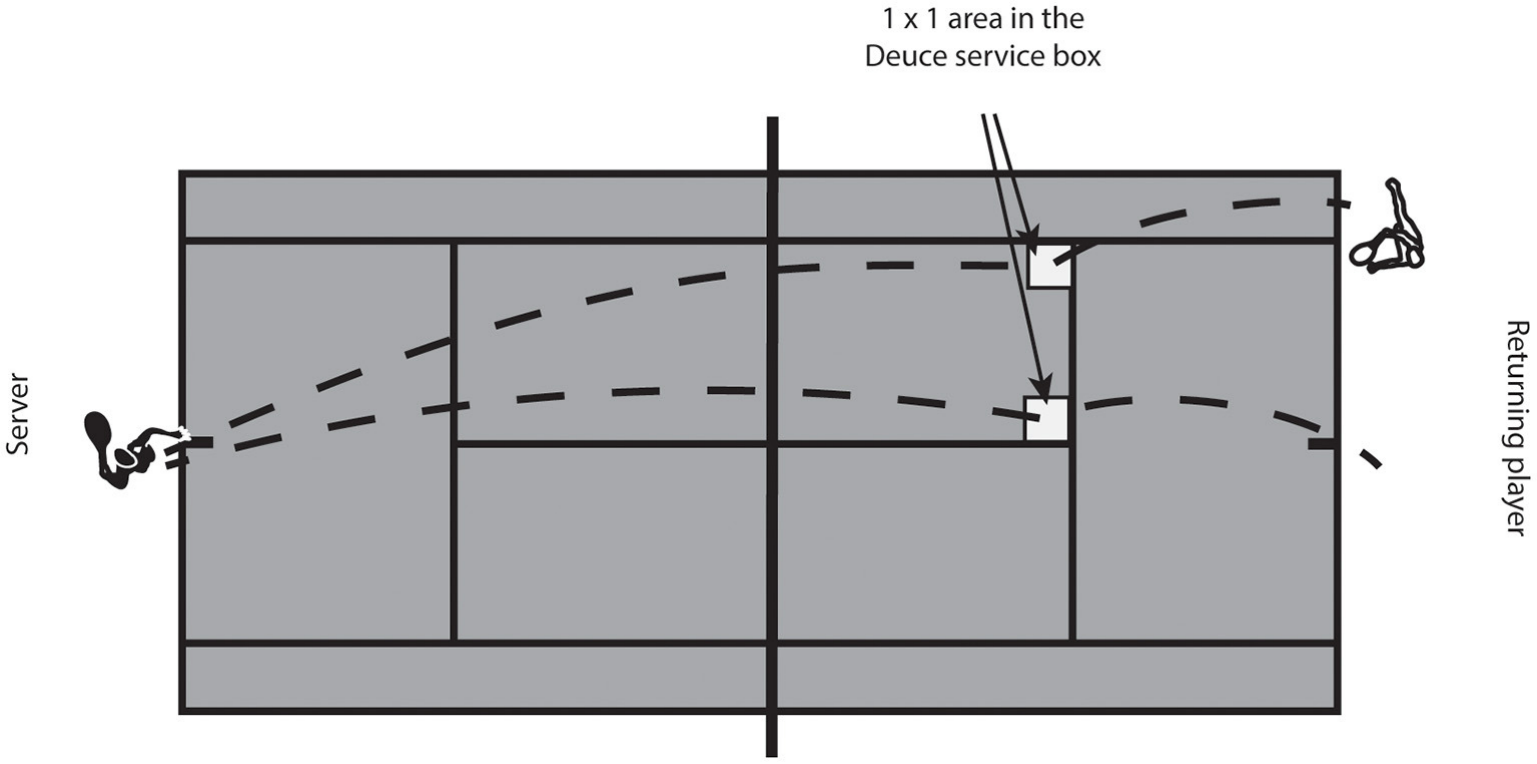
Experimental setup. The position of the server and returning player are presented, as well as areas where the server was required to position his serve in the returning player service box.

The experiment was conducted on a standard tennis hard court surface. Each participant performed ~90 returns on the same side of the court with the goal of scoring a point. Three servers switched intermittently after performing the first three serves (flat serves) into the 1 × 1 m square located in the left and right corners of the Deuce service box (Figure 1). Serves were distributed equally to each side but were semi-randomized. Participants and servers were instructed to continue the play on a successful return until one of the players scored a point. Three servers were recruited for each of the two groups, corresponding to their quality level. Players used their own racquets and strings during the testing procedure. The task was completed after 15 ± 9.7 min.

Two synchronized 50-Hz video cameras (Logitech C920, Logitech, Lausanne, Switzerland) were positioned at the edge of the court and recorded the movements of the server and the returning player as well as the ball flight. During returns, each returning player wore a 50-Hz eye tracker (Tobii Pro Glasses 2, Tobii, Danderyd, Sweden). An additional strap was used to fix the eye tracker to the head of a participant to prevent slippage. The head unit was connected *via* a USB cable to the recording unit, which was attached to the hip of a participant. Before the experiment, a single-target calibration routine was performed using the Tobii Pro Glasses Controller (Tobii Pro Glasses Controller, Tobii, Danderyd, Sweden). After calibration, the participant was instructed to direct his gaze at a 0.1 m target placed at the position of a server while standing on the opposite side of the court behind the baseline. If the gaze position did not overlap with the target, the calibration routine was repeated until sufficient accuracy was achieved. Before the start of the experiment, each participant performed a warm-up routine consisting of 15 returns to get adjusted to the glasses. None of the participants reported any discomfort while wearing the eye tracker during the experiment.

### Data Analysis

All returns were analyzed *post-hoc* by two nationally certified tennis coaches. They classified each return performance as follows: (i) tactically superior return, meaning that the server could not continue the play or had significantly reduced tactical options (usually the ball landed just next to the side-line or under the server), (ii) tactically inferior return, meaning that the server had more tactical options to choose from (usually a slower or lob ball), or (iii) an error by the returning player, defined as an out or a ball lending in the net. All errors made by the serving player were excluded from this study. The agreement between the two coaches in classifying the return performance was 96%. The returns where the agreement between the two coaches was not reached were excluded from further analysis. Twenty returns were randomly selected for each of the three return performance categories. This resulted in 720 returns in the national group and 300 returns in the professional group.

Eye-tracker data were analyzed using Tobii Pro Lab software (Tobii Pro lab 1.145, Tobii, Danderyd, Sweden). Eye-tracking data were filtered using a raw filter without gap-fill interpolation and with noise reduction both available in the Tobii Pri Lab software. To assess the gaze behavior during the movement of a server, serve actions were categorized into the following five phases based on major serve events that also determine the important time frames for the emergence of movement synergies of servers (Shafizadeh et al., 2019, 2020), which are defined by the organization of different body parts that work together to achieve a specific goal of the movement task and provide stability and flexibility of the system (Latash et al., 2007): (i) the preparation phase, starting 500 ms before and ending at the first observable upward movement of the tossing hand (preparation), (ii) the ball toss, ending at the instance the ball left the hand (ball toss), (iii) the windup phase, ending at an instance when the racket began to move upward, (iv) the hitting phase, ending when the racket contacted the ball, and (v) the follow-through phase, ending at an instance when the gaze of the participant started following the flying ball. In addition, 25 areas of interest (AOI) were defined according to the movement synergies present during the movement of servers (Jackson and Mogan, 2007; Shafizadeh et al., 2019) (Table 1), six in the first, nine in the second, three in the third, two in the fourth, and five in the fifth phase of servers movement. Two of the AOIs in the second phase were determined to be the areas (i.e., the tossing hand movement area and the area of ball release) where the specific movement is going to occur.

**Table 1:** Definition of areas of interest in specific phases of the movement of servers.

	**1st PSM**	**2nd PSM**	**3rd PSM**	**4th PSM**	**5th PSM**
Area of interest	Area surrounding server	Tossing hand	Area of hand—racket movement	Area of hand—racket movement	Tossing hand
	Diagonal server—returner	Tossing hand movement area	Ball contact area	Head and upper body	Hand—racket
	Ball bounce area	Ball release	Head and upper body		Head
	Tossing hand and upper body	Ball upwards flight			Upper body
	Racket hand	Back leg			Lower body
	Racket	Front leg			
		Server head			
		Upper body			
		Hips			

*Individual areas of interest are presented and the phases of the movement of a server they belong to; PSM, phases of server movement*.

The actual gaze positions (represented by a marker with a size of 0.73° of the visual angle, corresponding to 0.31 m at 25 m distance) were hand-mapped by an experimenter naïve with respect to the research question and the return performance category to a corresponding AOI in the corresponding phase. If the marker left the AOI with its edge, it was considered to have left the area where it was located in the previous sample. The procedure allowed the calculation of fixation durations in each AOI in milliseconds. These were defined as focal visual fixations that lasted longer than 100 ms and did not move outside the respective AOI (size between 0.72° and 2° of visual angle). The fixation duration to each AOI in each individual return was calculated and used for further analysis.

### Statistical Analysis

Data analyzes were performed using Orange data mining software (Orange 3.26.0, University of Ljubljana, Ljubljana, Slovenia). To analyze the relationships between gaze fixation duration in individual AOIs and categories such as individual server, returning player, or return performance category, the non-linear random forest machine learning approach was used (Liu et al., 2012). The duration of fixations in individual AOI during corresponding serve phases for both groups of participants were used as predictor variables, and the return performance category, returning player, or individual server as predicted classes. First, using Naïve Bayes, seven predictor variables were identified that allowed the highest prediction probability for the individual predicted classes identified *via* a nomogram (Zhang and Su, 2004; Shariat et al., 2009). The seven predictor variables were fed into the random forest machine learning algorithm to classify the data into specific subgroups for each of the predicted classes separately. To develop the machine learning classifier, the predictor variables from 1,020 returns (all players combined) were randomly split into five folds. Four folds were used for model training and cross-validated with the remaining fold, repeating the procedure for all folds. For the random forest, the number of trees was varied and the set of 13 trees with the split subset limit set to five enabled the highest accuracy of the model (Liu et al., 2012; Rigatti, 2017). The performance of the machine learning classifier for each data set was described by the area under the curve (AUC), classification accuracy (CA), sensitivity (true-positive rate), and specificity (false positive rate).

In the second step, two separate data sets were created for each group, and the procedure described above was repeated for the prediction of return performance, returning player, and individual server. By splitting the data sets, the specifics of each group could be examined.

Finally, for all three data sets (both groups and combined data from both groups), seven predictor variables were evaluated according to their importance in the machine learning classifier that the random tree method created. These were classified by fast correlation-based filter (FCBF), taking into account redundancy due to pairwise correlations between predictor variables (Lei and Liu, 2003). The first seven predictor variables that were >0.001 were included.

## Results

### Classification Accuracy for Both Groups Together

**Figure 2:**
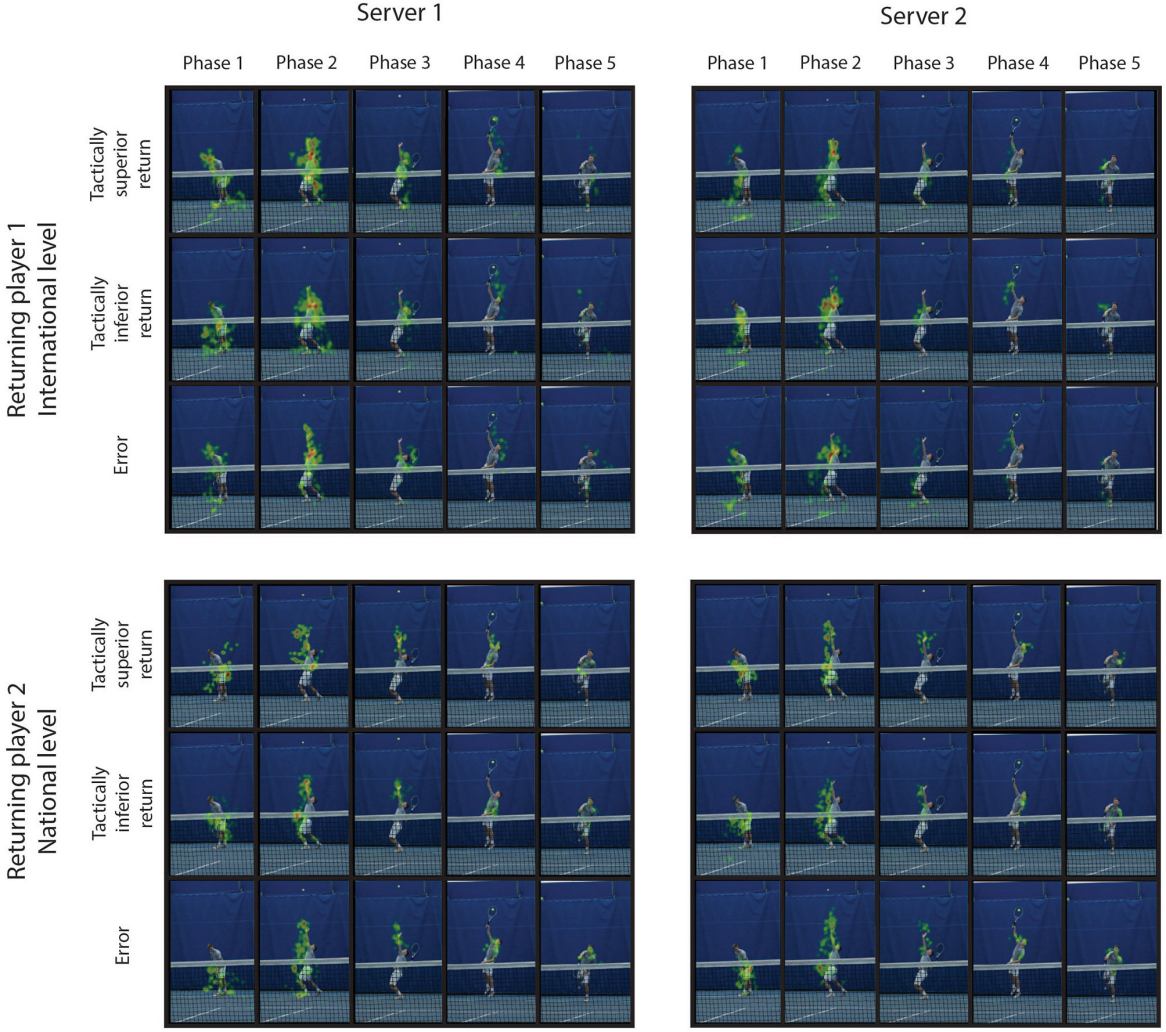
Heat maps show examples of visual behavior in two returning players. Examples of heat maps for two returning players (upper and lower set of heat maps) each belonging to a different group are presented. The left and right sets of heat maps represent visual fixation duration and location while observing two different servers. The upper row of pictures in each individual set of heat maps depicts visual fixation duration in each phase of the movement of a server at tactically superior returns, the middle row depicts tactically inferior returns, and the bottom row represents the returns resulting in error.

The performance of the random tree classifier is presented in Table 2. For the data set combining tennis players from both groups, the highest predictability was observed for the returning player followed by the classification of the return performance category, and the lowest predictability was observed for the individual server. In addition, the highest sensitivity was observed for the returning player and the lowest for the individual server classification. Similarly, the highest specificity was observed for the returning player classification and the lowest for the individual server classification. Examples of the visual behavior in two returning players are presented by heat maps in Figure 2.

**Table 2:** Performance of different classification models.

	**Both groups**				**National tennis players**				**International tennis players**			
	**AUC**	**CA**	**Se**	**Sp**	**AUC**	**CA**	**Se**	**Sp**	**AUC**	**CA**	**Se**	**Sp**
Server	0.686	0.215	0.201	0.214	0.834	0.453	0.577	0.526	0.901	0.575	0.575	0.575
Returning player	0.953	0.664	0.735	0.664	0.970	0.780	0.801	0.780	0.883	0.642	0.710	0.642
Return category	0.667	0.523	0.595	0.523	0.717	0.581	0.621	0.581	0.753	0.549	0.583	0.549

*AUC, area under the curve; CA, classification accuracy; Se, sensitivity; Sp, specificity are presented for classifying individual server, returning player, and return category for all returns combined and for returns made by national and international tennis players separately*.

### Classification Accuracy for the Group of National Players

Similar trends were observed in the data set consisting of only national level tennis players. The highest predictability was observed for the returning player classification, followed by the return performance category classification, and the lowest predictability was observed for the individual server classification. The highest sensitivity and specificity were observed for the returning player classification, followed by the return performance category classification and individual server classification.

### Classification Accuracy for the Group of International Players

For the international player group, the trend was somewhat different. The highest predictability was observed for the returning player followed by the individual server classification and the lowest for the return performance category classification. The highest sensitivity and specificity were observed for the returning player classification followed by the individual server classification and return performance category classification.

### Most Important AOI Attributes

Scores for individual predictor variables with FCBF exceeding 0.001 are presented in Table 3. Tossing hand movement area in the second phase of the movement of a server proved to be one of the most crucial predictors for classifying individual servers in both groups studied, especially in the national level tennis players (Table 3). Other predictor variables differed between predicting classes in both groups, with the international players showing the most important predictor variables in the first four phases of the movement of a server, and the national level players showed differences in all five phases.

**Table 3:** Classifiers best predicting the individual server and return performance category.

	**All**		**International tennis players**		**National tennis players**	
	**Classifiers**	**FCBF**	**Classifiers**	**FCBF**	**Classifiers**	**FCBF**
Server	2—Tossing hand movement area	0.132	2—Tossing hand movement area	0,125	2—Tossing hand movement area	0.281
	2—Ball upwards movement	0.075	4—Area of hand-racket movement	0.097	3—Ball contact area	0.128
	5—Lower body	0.075	1—Area surrounding server	0.060	5—Lower body	0.127
	1—Area surrounding server	0.061	4—Head and upper body	0.059	1—Racket	0.108
	1—Racket	0.058	3—Area of hand-racket movement	0.044	2—Ball upwards movement	0.092
	2—Hips	0.057			3—Area of hand-racket movement	0.092
	2—Ball release	0.049			1—Area surrounding server	0.091
Return category	2—Tossing hand movement area	0.132	2—Tossing hand movement area	0.050	3—Area of hand-racket movement	0.068
	2—Ball upwards movement	0.075	5—Lower body	0.047	1—Racket	0.056
	5—Lower body	0.075	4—Area of hand-racket movement	0.042	5—Lower body	0.046
	1—Area surrounding server	0.061	4—Head and upper body	0.034	4—Head and upper body	0.039
	1—Racket	0.058	2—Ball release	0.012	2—Back leg	0.014
	2—Hips	0.057	1—Racket hand	0.009	2—Ball release	0.008
	2—Ball release	0.049			4—Head and upper body	0.008
Returning player	2—Tossing hand movement area	0.183	2—Ball upwards movement	0.129	2—Tossing hand movement area	0.324
	2—Ball upwards movement	0.130	2—Tossing hand movement area	0.112	4—Area of hand and racket movement	0.217
	4—Area of hand and racket movement	0.121	1—Area surrounding server	0.111	2—Ball upwards movement	0.186
	1—Area surrounding server	0.072	4—Head and upper body	0.105	2—Upper body	0.124
	5—Lower body	0.067	5—Lower body	0.078	5—Lower body	0.100
	2—Upper body	0.067			3—Area of hand-racket movement	0.089
	3—Area of hand-racket movement	0.057			1—Area surrounding server	0.066

*Values of the fast correlation-based filter (FCB) are presented for seven most important predictor variables for classifying individual server, returning player, and return category for all returns combined and for returns made by national and international tennis players separately*.

The most successful predictor variables for the return performance category classification differed in the two observed groups of tennis players. The tossing hand movement area located in the second phase of the movement of a server for the international level tennis players and the area of hand-racket movement for the national level tennis players proved to rank highest between predictor variables (Table 3). International level tennis players tended to be classified based on a smaller number of AOI as in the national level tennis players. In general, the return performance category in the international level tennis players differed more by the length of visual focus duration directed to the tossing hand movement area in the second phase of the movement of a server and specific AOI in the fourth and fifth phases of the movement of a server as compared with national level tennis players who differed more in the amount of visual focus duration directed to the area of hand-racket movement in the third phase of the movement of a server and specific AOI in the fourth and fifth phases of the movement of a server.

As for classifying the returning player, the duration of visual focus of the international level tennis players differed more in observing the ball upward movement and tossing hand movement area in the second and third phases of the movement of a server (Table 3). In contrast, national level tennis players differed in visual focus duration in the tossing hand movement area during the second phase of the movement of a server and the area of hand and racket movement in the fourth phase of the movement of a server. In general, national level tennis players tend to differ from each other in their duration of focal visual attention in a higher number of AOI located in different phases of the movement of a server, which indicated higher variability between visual search strategies between national level tennis players.

## Discussion

In this study, the visual behavior during tennis serve return was investigated in relation to the returning player, individual server, and return performance category in two different groups of experts. The visual behavior was most strongly related to the returning player in both groups, indicating high interindividual variability as suggested by previous studies (Murray and Hunfalvay, 2017; Sáenz-Moncaleano et al., 2018). The second and third most successful classifications were for the return performance category and individual server, respectively, confirming our hypothesis that focal vision fixation duration at different AOIs was more related to the returning player than to other aspects such as individual server and return performance category. Interestingly, the individual server classification was low but more successful in the international group as compared with the national level tennis players. This suggests that the international players adapted visual search strategies to different servers more than the national level tennis players. The lowest ability to make predictions based on the duration and location of visual attention was observed for the return performance category. This was to be expected as serve return also depends on the observation of the ball flight phase (Sáenz-Moncaleano et al., 2018). In addition, seven or fewer AOIs from different phases of the movement of a server were identified that were best suited to differentiate between returning players, servers performing the tennis serve, and return performance categories.

The ability to classify an individual returning player suggests high interindividual variability and confirms findings from previous studies that participants use different visual search strategies (Button et al., 2011; Dicks et al., 2017). The availability of different sources of relevant visual cues and differences in the ability to interpret this information by individual performers could explain such differences between observers (Whiteside et al., 2013; Myers et al., 2017). This allows athletes to use different sources of visual information to perceive the movements of the same characteristics of opponents and consequently to vary their visual attention between repetitions and between individuals. This observation has been recently confirmed in field hockey goalkeepers, where different sources of visual information have been used by different goalkeepers all enabling successful saves, even after performing the same visual attention training intervention (Morris-Binelli et al., 2021).

Regardless of high interindividual variability, Alder et al. showed that visual search strategies in more skilled badminton players were highly related to the ability to attend to the most important visual information provided by the movements of an opponent (Alder et al., 2014). They showed that more skilled badminton players were able to focus on various kinematic cues that were better related to the trajectory of the ball and consequently were better able to make appropriate tactical solutions and shots. This was only partially observed in this study, with the visual behavior during the movement of a server being somewhat more strongly related to the return performance category in international players than in national level players. As suggested by other similar research, early information describing the movement of a server contributes significantly to online control of body movement and anticipation of ball flight direction and speed (van Soest et al., 2010; Müller and Abernethy, 2012; Triolet et al., 2013). This suggests that international tennis players in this study were more successful in using information from the movement of a server to predict the ball flight characteristics as their national counterparts. Our results add that a larger proportion of visual behavioral traits while observing movements of servers is related to intraindividual differences than to the return performance category, which is also in line with research performed by Morris-Binelli et al. (2021).

Interestingly, the observed classifications of the individual server were less accurate as for the returning players, especially among national-level tennis players. This may indicate that national-level tennis players in particular tend to use generic visual search strategies that are less attuned to the specifics of an individual server. In contrast, the visual search strategies of international level tennis players were more server-based, suggesting that the more experienced players are better able to adapt their visual search strategies to the specific visual cues of individual servers. However, these observations must be interpreted with caution. First, the sample size was small, particularly in the international tennis player group, as highly skilled international players are difficult to recruit. Second, an important limitation of this study was that differences between individual serving techniques were not examined. Therefore, future research should investigate whether the ability to adapt visual attention to the individual server is also related to other factors such as serve type, individual technique, and style and whether this adaptability affects interception performance.

The AOIs that were most important for classifying individual returning players when all participants were combined were in the first, second, fourth, and fifth phases of the movement of a server. Interestingly, the first two AOIs, namely, tossing hand movement area and ball upward movement, were also the most important predictors of return performance category and individual server. These results are consistent with the observations of Jackson and Mogan (2007), who pointed out the importance of ball toss for ball flight anticipation, as well as observations that important kinematic synergies are typical for these phases and can be specific for an individual server (Shafizadeh et al., 2019, 2020). Other AOIs located in the following phases of the movement of a server have been shown to contribute less to the classification of returning player and individual server. These observations are partially consistent with the results of studies performed in other interceptive tasks, where the importance of visual attention increases as the observer approaches closer to the movement initiation or interception (Button et al., 2011; Navia et al., 2017), such as movement initiation of returning players in this study. After the first two phases, the movement of a returning player is already initiated and begins to rely more on online control using information from ball flight characteristics.

The duration of focal vision fixation on movements of the tossing hand and the upward movement of the ball also differ significantly between the return performance categories. This suggests that players may interpret this phase of server action differently, which strongly influences the performance of the return, which is in line with the latest research (Morris-Binelli et al., 2021). As could be speculated on the above rationales, this phase is highly related to the first movement initiation of the returning player toward the interception point. If this movement initiation is delayed, the return performance might be compromised.

In the national level group, players also differed in visual attention duration focused on lower body parts such as hips and legs in the first three phases of the movement of a server. This can be better interpreted by including the results from the return performance category classification. The comparison of the two groups shows that in the international group, hand and racquet movements during all phases are the primary AOIs that relate to the return performance category. In contrast, in the national level group, visual attention duration to other AOIs, rather than hand and racquet movements, is related to the return performance category. Since more AOIs are important in the national level group for discriminating the return performance category, it could be speculated that their visual attention is directed to more different sources of visual information, which could reduce the time spend observing more important movements of the opponent. A limitation of this study was that the specific effect that each AOI had on classification was not examined. This would be of importance, as some AOIs that were found to be important classifiers could have a negative effect on return performance. As suggested by the studies on quiet eye phenomena, more skilled athletes have fewer saccades and longer fixations to the most relevant AOI (Lebeau et al., 2016; Gonzalez et al., 2017). Applying these observations to this study, one could hypothesize that the longer duration of visual attention focused on the lower body of an opponent negatively affects the return performance.

Our results also show that the international players returning the serve also observe the area next to the tossing hand and the ball release area in the second phase of the movement of a server. These two areas were defined as the areas where the hand and the ball will be located in the upcoming moments. This visual behavior could be interpreted as anticipatory visual behavior or as more efficient buffering between visual information pickup and motor response. As suggested by Connor and Knierim (2017), the movement of focal vision to the areas of anticipated events suggests the anticipation of opponent movement and prompts recall of the anticipated action from visual memory. Being primarily present only in the international group, these tennis players might show better anticipation of the movement of a server, which could improve their movement response. Similar visual behavior has been reported by other studies (Land and Furneaux, 1997; Vickers and Adolphe, 1997; Furneaux and Land, 1999; Land and McLeod, 2000; Qian et al., 2019). These authors proposed that the earlier movement of focal vision relative to observer movement is enabled by visual working memory, which integrates information that cannot be observed simultaneously. As these studies show, more skilled performers have a higher buffering capacity, which allows for the integration of a greater amount of information. This could have important implications for responding to such complex visual stimuli as the tennis serve. Since noisiness of the movements of an opponent and a ball contains different relevant and irrelevant information, a higher visual memory capacity could be beneficial to include only the most relevant information. Based on the results of this study, it could be speculated that more skilled participants could have had superior visual memory capacity, besides more efficient information pickup strategies.

The classification of the return performance category suggests that the fourth and fifth phases of the movement of a server are important for success, but to a lesser extent. As our results suggest, the more experienced players are better able to extract information from the racquet upward movement, ball contact, and follow-through movement. According to van Soest et al. (2010), the final ball contact and the follow-through movement are characterized by funneling of the end effector (hand and racquet). Funneling represents a low degree of intertrial variability and is biomechanically highly representative of ball flight characteristics and therefore provides important visual cues for the tennis player returning the serve. As visual attention is more important at this stage for national level tennis players, it could be speculated that they rely more on online motion adaptation, possibly compensating for less efficient anticipation of ball flight characteristics during the movements of a server.

Overall, the results of this study suggest that a large interindividual difference in visual attention during serve return exists between tennis players. However, visual search strategies in more experienced tennis players may also adapt to the specific opponent. This is consistent with previous research showing more efficient anticipation based on the initial opponent movement. However, the results of this study add that more experienced athletes are better not only at predicting ball flight characteristics but also able to adapt to the specific constraints presented by an individual opponent.

These findings additionally present important implications for training tennis serve return. Bonato et al. (2020) reported on the positive effects of visual attention training on specific aspects of tennis serve return performance in junior tennis players. Findings from our research show that future training studies should additionally use tennis serve specific visual information, primarily focused on ball toss and ball vertical flight observation, which enable more efficient anticipation and movement initiation. This could be achieved *via* video or model-based observations or on-court training. Improved ability to recognize hand, ball, and racquet movement patterns could enable more efficient buffering of visual information, which could enable processing of other important visual cues that could altogether provide more reliable prediction of the ball flight trajectory. Moreover, such training routines should introduce variable servers and serve types to learn how to apply basic principles of visual search strategies to different opponents and serve types.

## Data Availability Statement

The raw data supporting the conclusions of this article will be made available by the authors, without undue reservation.

## Ethics Statement

The studies involving human participants were reviewed and approved by Slovenian Committee for Medical Ethics. The patients/participants provided their written informed consent to participate in this study.

## Author Contributions

JR and ZM: conceptualization, data analysis, investigation, resources, writing—review and editing, visualization, and supervision. JR: methodology and writing—original draft preparation. Both authors have read and agreed to the published version of the manuscript.

## Conflict of Interest

The authors declare that the research was conducted in the absence of any commercial or financial relationships that could be construed as a potential conflict of interest.

## Publisher's Note

All claims expressed in this article are solely those of the authors and do not necessarily represent those of their affiliated organizations, or those of the publisher, the editors and the reviewers. Any product that may be evaluated in this article, or claim that may be made by its manufacturer, is not guaranteed or endorsed by the publisher.
